# Climate change impact on future Egypt’s wind energy: a CMIP6-based assessment of power output

**DOI:** 10.1038/s41598-026-58105-w

**Published:** 2026-06-24

**Authors:** Mohammed Magdy Hamed, Mohamed Tarek Sobh, A. R. El-Mallawany, A. O. Elgharib

**Affiliations:** 1 Construction and Building Engineering Department, College of Engineering and Technology, Arab Academy for Science, Technology and Maritime Transport (AASTMT), B2401 Smart Village, Giza, 12577 Egypt; 2 Mechanical Engineering Department, College of Engineering and Technology, Arab Academy for Science, Technology and Maritime Transport (AASTMT), B2401 Smart Village, Giza, 12577 Egypt; 3 Basic and Applied Science Department, Arab Academy for Science, Technology, and Maritime Transport (AASTMT), B2401 Smart Village, Giza, 12577 Egypt

**Keywords:** Wind energy, Wind turbines, ERA5-land, Shared Socioeconomic Pathways (SSPs), Global Climate Models (GCMs), Kling-Gupta Efficiency (KGE), Climate sciences, Environmental sciences

## Abstract

Egypt possesses substantial potential for renewable energy generation, prompting heavy national investments to increase the share of wind power in its overall energy portfolio. Consequently, it is crucial to evaluate the long-term vulnerability of future wind energy production to climate change. This study fills a critical gap in regional climate-energy modelling by providing a novel quantification of turbine-specific capacity ratios across four Shared Socioeconomic Pathways (SSP1-2.6, SSP2-4.5, SSP3-7.0 and SSP5-8.5). Through a comparative assessment of 23 CMIP6 Global Climate Models (GCMs), EC-Earth3-Veg, EC-Earth3, and CESM2-WACCM were identified as the most reliable models against historical ERA5-Land data using the Kling-Gupta Efficiency (KGE) metric, followed by Quantile Mapping for bias correction of both historical and future scenarios. Evaluating nine wind turbine models (T1–T9) revealed that T1 and T2 maintained the highest historical capacity ratios, peaking at 68.0–76.5% and 59.5–68.0%, respectively. By 2100, meteorological projections indicate a regional warming trend coupled with a decrease in mean wind speed; notably, the high-emission SSP5-8.5 scenario projects the highest mean temperature (28 °C) and lowest mean wind speed (3.8 m/s). Despite these declines, future projections for T1 and T2 indicate resilient power generation and localized increases in strategic locations, such as Ras Ghareb and southern Egypt, particularly under the SSP2-4.5 scenario. Ultimately, these findings provide essential data-driven insights for energy planners to optimize turbine selection and site development, ensuring the long-term resilience of Egypt’s wind energy infrastructure.

## Introduction

The transition from fossil fuels toward renewables is occurring and accelerating due to technological advancements, policy, and decreasing costs^1^. Solar, wind, and battery storage are now substantially less expensive than coal and gas^2–4^, prompting a decisive shift in international energy planning toward sustainable infrastructure. Wind turbines are paving the way to worldwide systems of renewable energy in which wind kinetic energy is converted into electricity^5^. Present-day horizontal-axis wind turbines (HAWTs) are the most common design, featuring three aerodynamically designed blades optimised to capture wind energy efficiently^5,6^. To maximise this potential, research and development are currently focused on enhancing efficiency, lifespan, and cost-effectiveness^7^. Advances in material technology are delivering stronger, lighter materials for blades, enabling larger-diameter turbines^8,9^ with greater capacity to harvest wind energy^6^. Furthermore, direct-drive generators without gearboxes, along with digital technologies such as high-calibre sensors, data analytics, and artificial intelligence, are increasingly employed to optimise performance and maximise grid integration^5,6,10^.

On a national scale, this transition mitigates global emissions while reducing economic vulnerability to volatile fossil fuel markets^11–13^. In alignment with the United Nations Sustainable Development Goals (SDGs), the Egyptian government aims for 42% of electricity generation to come from renewable sources by 2035 as part of its Vision 2030^14^. Unlike many countries, Egypt has vast, uninhabited desert land with high potential for wind energy^15^. Wind turbine installations have been heavily concentrated in the Gulf of Suez region, particularly around Zaafarana^16^, where average wind speeds exceed 10 m/s^17^. While high ambient temperatures and sandstorms present challenges that increase maintenance costs^18^, this wind energy potential acts as a critical advantage for the future evaluation and production of green hydrogen in Egypt^19^. Furthermore, expanding wind energy into southern Egypt offers a practical pathway to build sustainable communities in line with SDG 11^20^.

However, because wind turbines rely entirely on local climate, their long-term success is highly vulnerable to a changing climate^21^. To ensure these future projects are viable, it is essential to project future climate change and systematically evaluate how shifting patterns will alter available wind power density^22^. The sixth phase of the Coupled Model Intercomparison Project (CMIP6), coordinated by the World Climate Research Program, provides the necessary framework by simulating historical and future climate changes under the influence of greenhouse gases^23,24^. CMIP6 utilises Shared Socioeconomic Pathways (SSPs) such as SSP1-1.9, SSP1-2.6, SSP2-4.5, SSP3-7.0, and SSP5-8.5, which combine underlying socioeconomic narratives with corresponding Radiative Forcing levels in the year 2100 to quantify resulting warming levels and societal mitigation capacities^25^.

While previous studies^26–28^ have analysed wind speed trends over North Africa, recent literature focuses on the future of wind energy across Africa^29^. The capacity factor under SSP2-4.5 and SSP5-8.5 scenarios for 2041–2070 and 2071–2100 has been studied. The high wind potential in Northern, Eastern, and Southern Africa suggests that these regions could support substantial wind projects. Although certain Northern locations in Africa show minimal or negative changes, indicating regional vulnerability that necessitates careful site selection and potentially alternative renewable energy strategies. Climate data from the past four decades was utilised to model expected shifts in tropospheric circulation for the Middle East and North Africa (MENA) region from 2066 to 2100^30^. The findings illustrate the impact of atmospheric composition on radiation, circulation, convection, and rainfall. Different parts of Egypt are expected to experience different trends of changes in the annual wind speed^31^. The wind speed is expected to decrease in the northern coastal zone, while increasing in other parts under the RCP 8.5 scenario by the year 2065, compared to the baseline climate period. A critical gap remains in identifying which specific turbine technologies and geographical locations in Egypt will remain productive until the end of the 21st century. Currently, the wind turbines deployed across Egypt are predominantly medium-scale (2 MW)^32,33^. However, as the future of wind energy in Egypt evolves, assessing the potential of large-scale wind turbines under changing climatic conditions is vital^31^.

To address this gap, the present study contributes a novel framework that bridges global climate pathways with local-scale energy production viability. The present study evaluates the future wind energy potential in Egypt (particularly within the highly productive Red Sea and Gulf of Suez regions) by assessing the impact of climate change on wind speeds and temperatures through the end of the 21st century. The methodology leverages historical ERA5-Land reanalysis data alongside 23 Global Climate Models (GCMs) from CMIP6, utilising the Kling–Gupta Efficiency (KGE) metric to select the three most accurate models and applying Quantile Mapping (QM) to eliminate systematic biases. These corrected datasets are then projected across four SSP scenarios (SSP1-2.6, SSP2-4.5, SSP3-7.0 and SSP5-8.5) to project near and far future meteorological conditions. Wind speeds and air densities were used to calculate the extractable power outputs to evaluate the capacity ratios of nine different wind turbine models (ranging from 0.1 MW to 15 MW), aiming to identify the optimal technologies and sites for Egypt’s future large-scale wind power development.

## Study area and data sources

### Study area

**Fig. 1:**
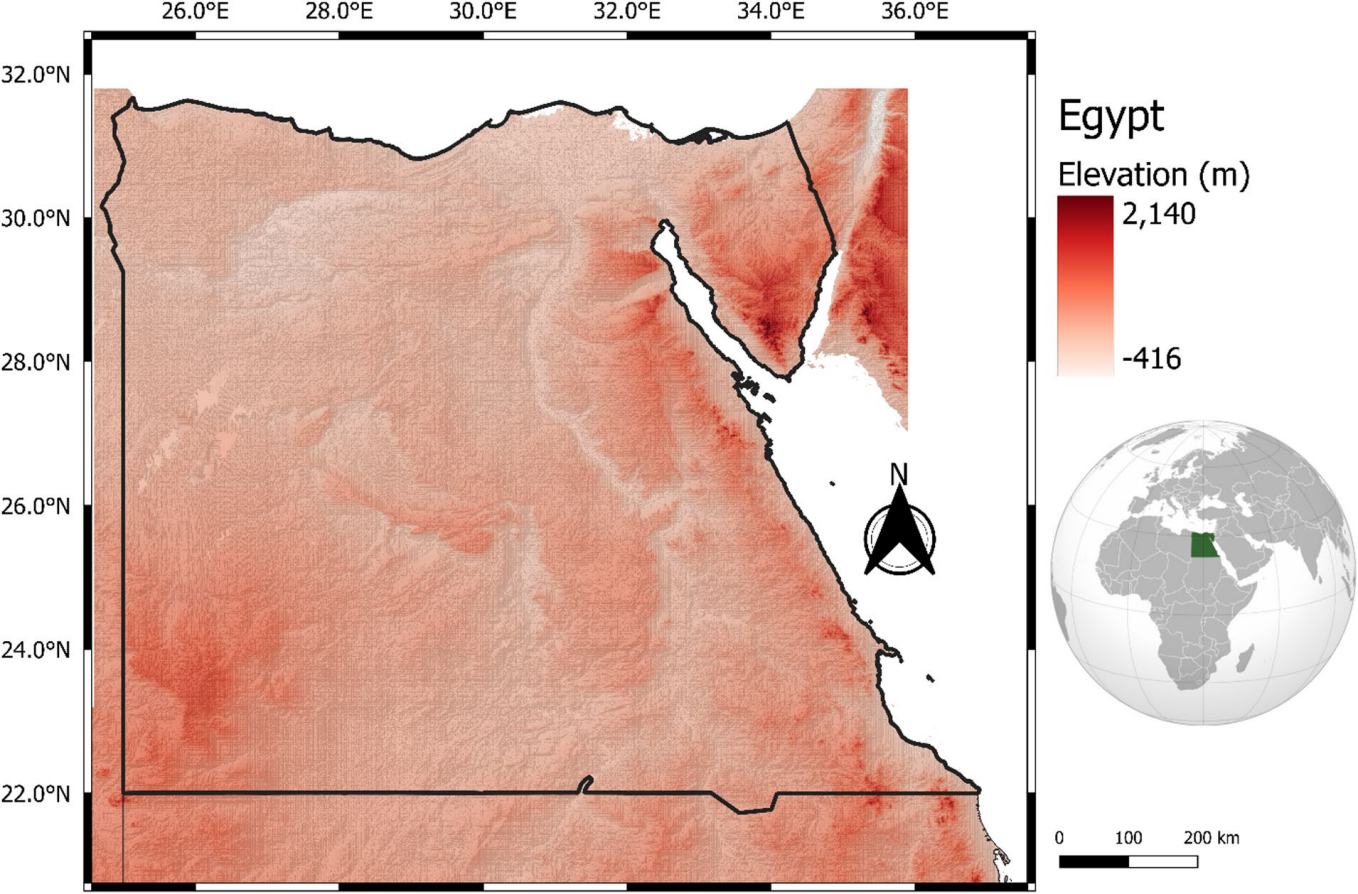
Study area map.

Egypt is in North Africa, as shown in Fig. 1, and lies approximately between latitudes 22 and 32 °N and longitudes 25 and 37 °E, bordered by the Mediterranean Sea to the north and the Red Sea to the east, connected by the Suez Canal^34^. The country is predominantly an arid, hot desert environment, characterised by a climate where minimum air temperatures typically range from 2.5 °C to 26.3 °C and maximum air temperatures from 16.6 °C to 41.0 °C, with minimal precipitation during the four seasons. The Nile River, the world’s longest, flows northward to create the fertile Nile Delta, effectively dividing the country into the vast Western Desert and the Eastern Desert. Remarkably, for wind energy estimation and projection^35^, the Red Sea coast and Gulf of Suez region present a globally exceptional wind resource. High-potential zones, such as the area around Ras Ghareb, have a significant annual mean wind speed consistently ranging between 7 m/s and 12 m/s. Furthermore, the wind resource is most consistent and powerful during the summer months, making this region the paramount focus for large-scale wind turbine power development^36^.

### Data collection

Due to climate change, wind speed and temperature will be affected in the coming decades^21^. In the present study, historical data from 1975 to 2014 of wind speed and temperature were compared with future data for the near and far future, covering the periods 2020 to 2059 and 2060 to 2099, respectively. The historical data are provided by ERA5-Land, while the future data are provided by GCMs, 23 GCMs used from CMIP6, presented in Table 1.

#### ERA5-land

ERA5-Land provides gridded data with a Horizontal resolution of 0.1° × 0.1° (9068 grid points)^37^. The data is available from 1950 to the present with hourly resolution. A total of 240 atmospheric variables are available for different pressure levels. ERA5-Land provides both the u and v components of wind speed, which can be combined to yield the magnitude and direction of the horizontal wind speed at 10 m. In addition, it provides the air temperature above land. This climate data supports scientists and software developers in developing interactive applications with real-world use cases.

#### Global Climate Model (GCM)

GCM is a mathematical model that simulates the Earth’s climate system, including the atmosphere, oceans, land surface, ice, and radiative forcing^38^. The main target is to simulate the historical climate and understand future climate scenarios and their impacts on ecosystems. Resolution, uncertainty, and computational complexity are the three main limitations of GCMs^39^. An uncertainty analysis needs to be addressed before creating the model. The initial variant label r1i1p1f1 for CMIP6 was selected to ease the evaluation procedure. Monthly historical (1975–2014) and future projection (2020–2099) Tmean, uas, and vas were used for the assessment. The simulations can be obtained from https://esgf-ui.ceda.ac.uk/search accessed on 1 June 2025.

**Table 1:** GCMs used from CMIP6.

No.	Model abbreviation	Resolution	Country	Variable used
1	ACCESS-CM2	1.87°×1.25°	Australia	Tas, uas, and vas
2	ACCESS-ESM1-5	1.87°×1.25°	Australia	
3	CanESM5	2.79°×2.81°	Canada	
4	CESM2-WACCM	0.94°×1.25°	USA	
5	CMCC-CM2-SR5	1°×1°	Italy	
6	CMCC-ESM2	0.94°×1.25°	Italy	
7	EC-Earth3	0.35°×0.35°	Europe	
8	EC-Earth3-Veg	0.35°×0.35°	Europe	
9	FGOALS-g3	2°×2°	China	
10	GFDL-CM4	1°×1.25°	USA	
11	GFDL-ESM4	1°×1.25°	USA	
12	IITM-ESM	1°×1°	India	
13	INM-CM4-8	2°×1.5°	Russia	
14	INM-CM5-0	2°×1.5°	Russia	
15	IPSL-CM6A-LR	2.5°×1.27°	France	
16	KACE-1-0-G	1.88°×1.25°	Republic of Korea	
17	MIROC6	1.41°×1.4°	Japan	
18	MPI-ESM1-2-HR	0.94°×0.94°	Germany	
19	MPI-ESM1-2-LR	1.87°×1.86°	Germany	
20	MRI-ESM2-0	1.12°×1.12°	Japan	
21	NorESM2-LM	2°×2°	Norway	
22	NorESM2-MM	1.25°×0.94°	Norway	
23	TaiESM1	0.9°×1.25°	Taiwan	

## Methodology

**Fig. 2:**
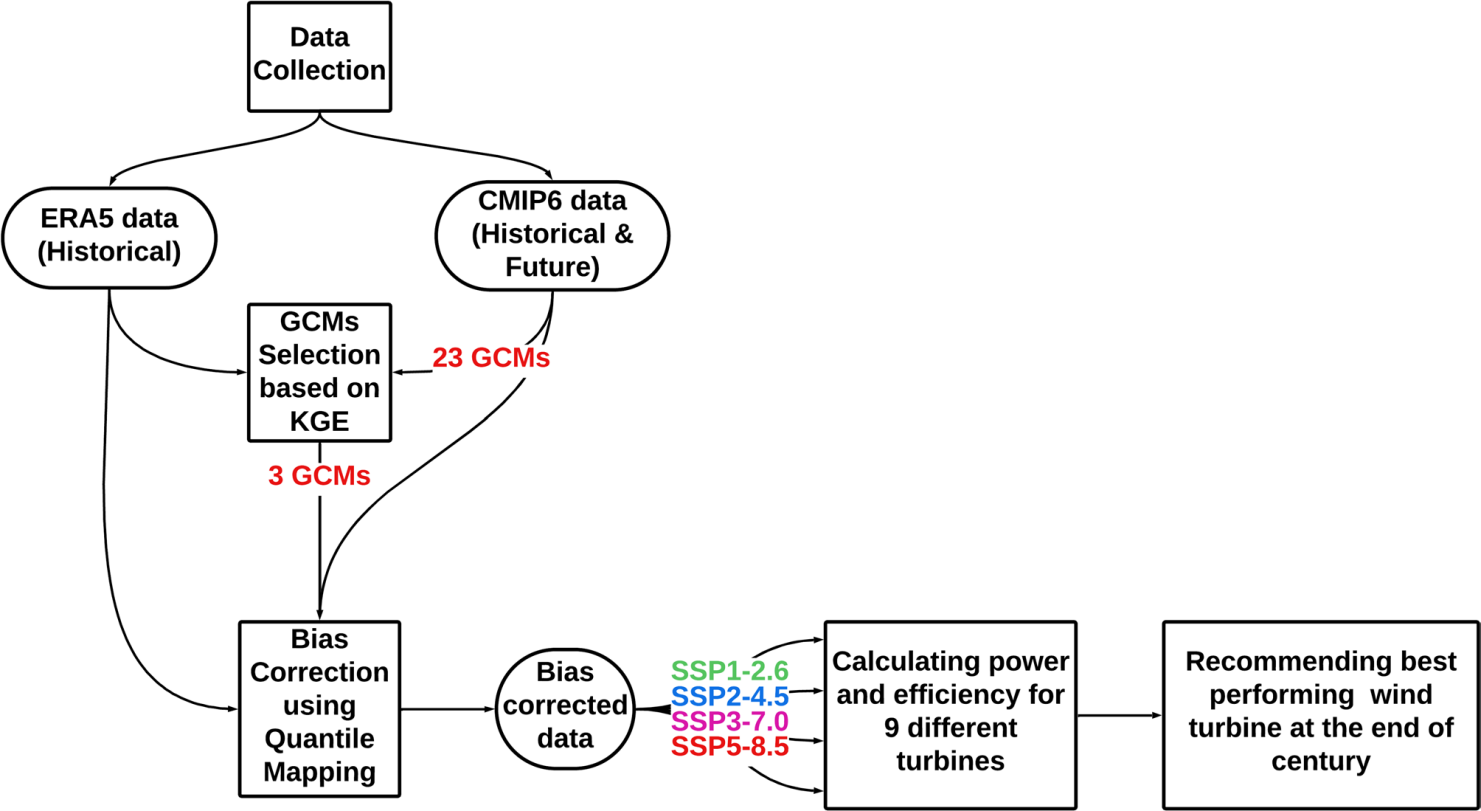
Flowchart of this study’s methodology.

The methodology for projecting the future of wind energy in Egypt, as summarised in the flow chart (Fig. 2), began with Data Collection utilizing two sources: the reference ERA5-Land reanalysis data for historical meteorological (temperature and wind speed) and CMIP6 GCM data, which provided both historical runs and future climate projections. To select the most reliable models, 23 GCMs were initially compared to ERA5-Land. The study attempted to minimise error by downscaling all CMIP6 models to the ERA5-Land spatial resolution using bilinear interpolation and selecting the best-performing models using the Kling–Gupta Efficiency (KGE) metric. The KGE metric was employed to rigorously evaluate the models based on their ability to replicate the historical temperature and wind speed distributions. This process resulted in the selection of three top-performing GCMs to be carried forward. Despite their strong performance, a subsequent bias correction step was necessary; this was executed using the Quantile Mapping (QM) technique to adjust the GCM outputs (historical and future), ensuring the historical wind speed and temperature data closely align with the statistical properties of the observed ERA5-Land data before being used for power estimation.

The bias correction was done for all SSPs, representing the full spectrum of climate futures: SSP1-2.6 (lowest emissions), SSP2-4.5 (stabilisation), SSP3-7.0 (high emissions), and SSP5-8.5 (highest emissions). The projected wind speed and temperature values for each of these four scenarios were systematically applied to calculate the output power and efficiency for a total of nine different wind turbines. The central objective of this comprehensive approach is to accurately predict the state of wind energy in Egypt by the end of the century. The final stage involves analysing the performance results across all scenarios and turbines to confidently recommend the best-performing wind turbine and suggest locations for future large-scale energy development in Egypt. Future wind power projections were calculated using the Multi-Model Ensemble (MME) mean of the three selected GCMs.

### Kling–Gupta Efficiency (KGE)

The statistical measure KGE^40^ compares simulated and observed data by accounting for multiple aspects of simulation performance. KGE is a function of ($\:\mathrm{r}$) the linear correlation between observations and simulations, (α) the measure of variability, and bias (β), as shown in Eq. 1. The variability is the ratio of the simulation standard deviation to the observed standard deviation. Also, bias is the ratio of the simulation mean to the observation mean. KGE was developed to overcome the limitations of other metrics, such as Nash-Sutcliffe Efficiency (NSE), by providing a more balanced assessment of model performance. KGE of 1 indicates high agreement between both simulation and observation^41^, while KGE values in the range of 0 to 0.5 indicate poor agreement^42^.

1 $$\:\mathrm{K}\mathrm{G}\mathrm{E}=1-\sqrt{{\left(\mathrm{r}-1\right)}^{2}+{\left({\upalpha\:}-1\right)}^{2}+{\left({\upbeta\:}-1\right)}^{2}}$$

### Bias correction

Based on systematic errors, the GCM outputs are not used directly^43^. The error is due to several factors, including low spatial resolution, simplified thermodynamics and physical processes, and incomplete knowledge of the climate system. Therefore, the raw historical and future climate model outputs must be bias-corrected to better fit the observed data. The Quantile Mapping (QM) technique removes systematic bias in the GCM simulations for both historical and future GCMs. The errors in the historical period will be the same in the projected period^44^.

### Wind power

The ERA5-Land provides the wind speed and temperature for 9068 nodes across Egypt at an elevation of 10 m. The wind speed magnitude V(Z) at a height Z is calculated by Eq. 2 for each node. Where u and v are the wind velocities (m/s) in the x direction and y direction, respectively. The boundary layer of the atmosphere is affected by turbulence at all altitudes, and topographical variation of the change with height and surface roughness. As a result, the average speeds increase with increasing altitude. All values of the wind speeds V(Z_R_) (m/s) were calculated at different heights Z_R_ (m) while considering the tower height and roughness height Zo (m) by Eq. 3. The density of air (ρ) was calculated based on the temperature of the air and the elevation of the turbine above mean sea level, as in Eq. 4. The height of the tower, as shown in Table 2, was considered while calculating the air density. Three-bladed upwind Horizontal Axis Wind Turbines (HAWT) are used in the present study. The maximum estimated extracted power P_tmax_ (W) by each turbine is calculated using Eq. 5. The estimated output power is calculated while considering the Betz limit. The ratio of the maximum available power to the rated power of the wind turbine was used to calculate the capacity ratio as shown in Eq. 6.

2 $$\:\mathrm{V}\left(\mathrm{Z}\right)=\:\sqrt{({\mathrm{u}}^{2}+\:{\mathrm{v}}^{2}\:)}\:\:\:$$

3 $$\:\mathrm{V}\left({\mathrm{Z}}_{\mathrm{R}}\right)=\mathrm{V}\left(\mathrm{Z}\right)\left(\frac{\mathrm{ln}\left(\frac{{\mathrm{Z}}_{\mathrm{R}}}{\mathrm{Z}\mathrm{o}}\right)}{\mathrm{ln}\left(\frac{\mathrm{Z}}{\mathrm{Z}\mathrm{o}}\right)}\right)$$

4 $$\:{\uprho\:}=\:\frac{353.049}{T}{e}^{(-0.034\:\frac{elev}{T})}$$

where T is the temperature (°K), and elev is the elevation of the turbine above mean sea level (m).

5 $$\:{\mathrm{P}}_{\mathrm{t}\mathrm{m}\mathrm{a}\mathrm{x}}=\frac{1}{2}\:{\uprho\:}\:{\mathrm{A}}_{\mathrm{T}}\:{\mathrm{V}}^{3}\frac{16}{27}$$

where, $\:{P}_{tmax}$ is the maximum power available (W), $\:{A}_{T}$ is the cross-sectional area (m^2^), and V is wind speed (m/s).

6 $$\:C=\frac{{P}_{tmax}}{{P}_{R}}$$

where $\:C$: Capacity ratio (%), $\:{P}_{tmax}$ is the maximum power available (W), and $\:{\boldsymbol{P}}_{R}$: rated power of wind turbine (W).

**Table 2:** Characteristics of the horizontal axis wind turbine (HAWT).

Name	Power (MW)	H (m)	*R* (m)
T1	15	166	135
T2	10	140	96.5
T3	5	100	65
T4	2	80	40
T5	1.5	80	38.5
T6	1	60	27.1
T7	0.75	50	23.5
T8	0.225	40	14.5
T9	0.1	24	10

## Results

**Fig. 3:**
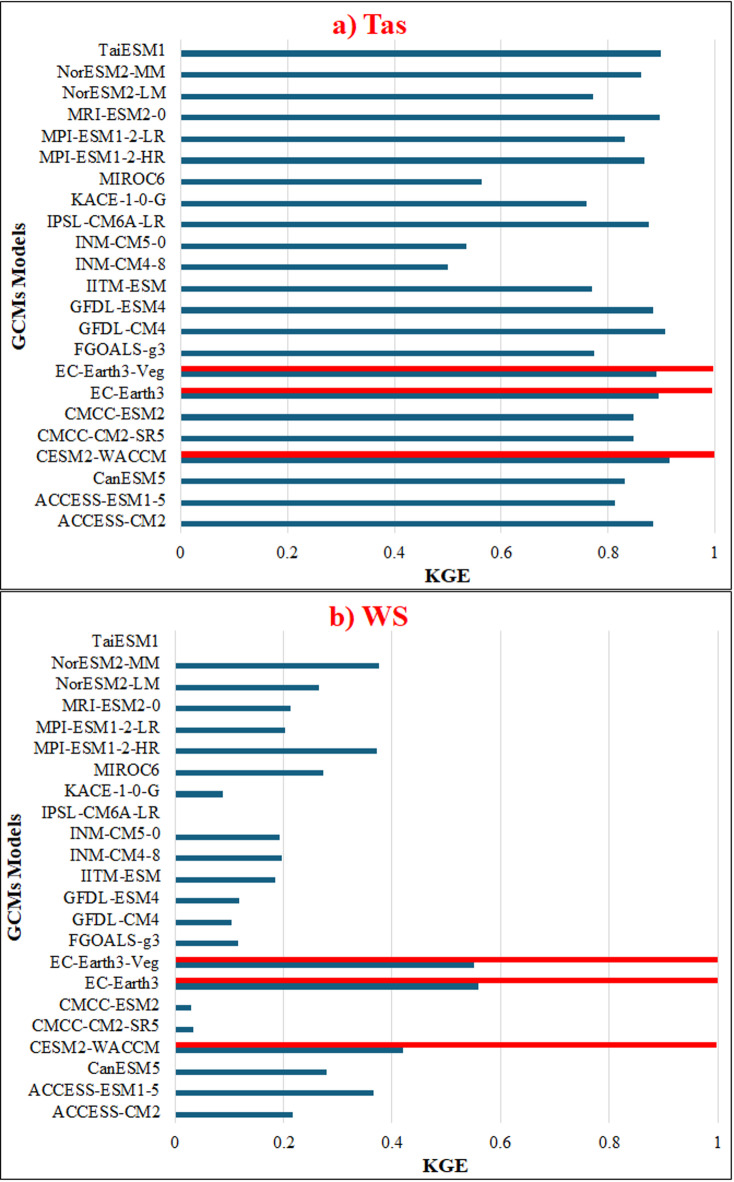
Global Climate Model selection compared to ERA5-Land data in historical (1975 to 2014) using the Kling–Gupta efficiency robust single metric.

Figure 3 illustrates the KGE results before and after bias correction by blue and red bars, respectively. Three GCMs were selected for bias correction as the best performing models in both temperature and wind speed, namely EC-Earth3-Veg, EC-Earth3, and CESM2-WACCM. The majority of the KGE values of the temperature are in the range from 0.7 to 0.9 when applying the 23 GCMs. The values of the KGE for EC-Earth3-Veg and EC-Earth3 while considering the wind speed were in the range from 0.5 to 0.55, and 0.43 for CESM2-WACCM. The KGE values of the wind speed for the rest of the models were less than 0.4. Then, a bias correction was applied to the three selected models. The output data were compared to that of ERA5-Land again. This is indicated by the red bars shown in Fig. 3 for both the temperature and wind speed. The KGE results reached a value of 0.99 after bias correction.

**Fig. 4:**
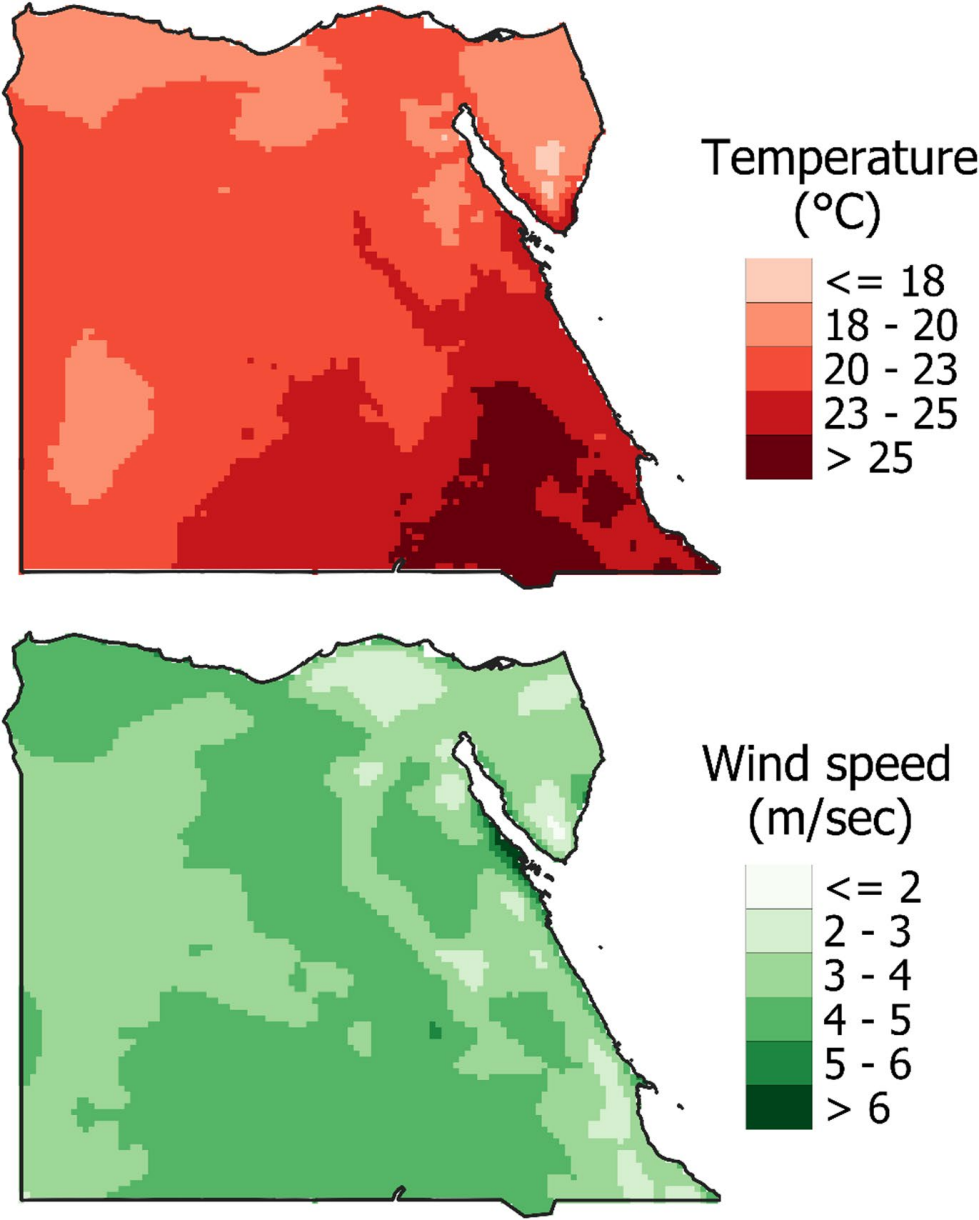
Spatial distribution of mean temperature (°C) and wind speed (m/s) across Egypt from 1975 to 2014.

Figure 4 represents the spatial distribution of mean temperature (°C) and wind speed (m/s) across Egypt. The historical data of both temperature and wind speed play a significant role in predicting the future of wind energy. The minimum and maximum values of the temperature across Egypt are found in the south of Sinai and the south of Egypt, respectively. The mean temperature is greater than 25 °C across the south-east of Egypt and in the range of 18 to 20 °C across the north-west of Egypt. The mean wind speed is in the range of 4 to 5 m/s across the Nile River and in the north-west and south of Egypt. The highest values of wind speeds are found in Ras Gharb, with more than 6 m/s.

**Fig. 5:**
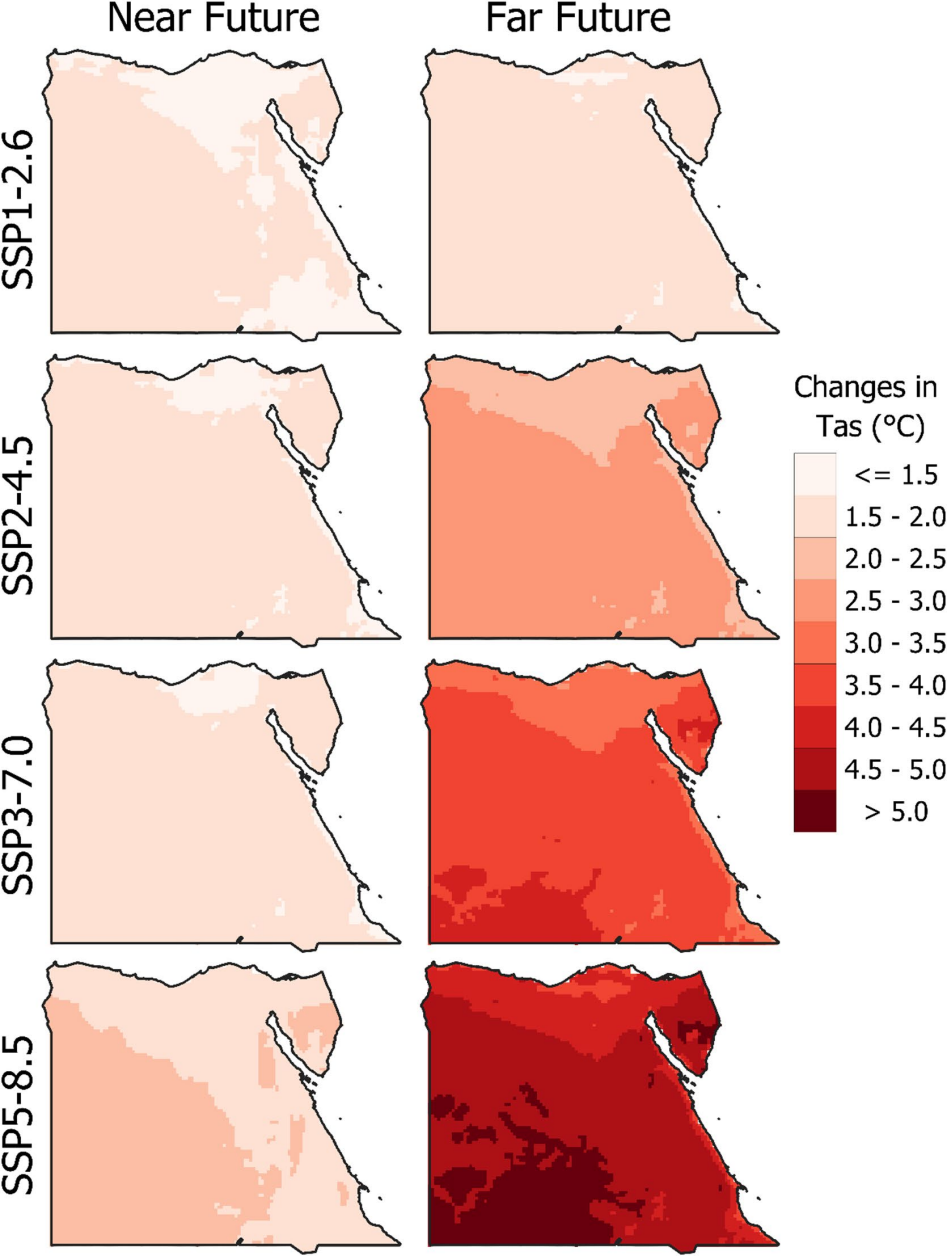
Absolute changes in mean MME temperature for both the near (2020 to 2059) and far future (2060 to 2099) from historical values.

The absolute changes in temperature for both the near and far future compared to historical values are shown in Fig. 5. Each row shows the absolute change in temperature while applying different SSPs. In the near future, the temperature change while applying SSP1-2.6, SSP2-4.5, and SSP3-7.0 is 2 °C across most of Egypt. The temperature change reached 2.5 °C while applying SSP5-8.5. Although there is no remarkable temperature change while applying the four different SSPs in the near future, the change in temperature is remarkable in the far future period. The temperature change across Egypt in the far future is 2 °C, 2.5 °C, and 3 °C while applying SSP1-2.6, SSP2-4.5, and SSP3-7.0, respectively. The change in temperature while considering SSP5-8.5 is 4.5 °C in the north of Egypt and increases gradually till it reaches 5.5 °C in the South.

**Fig. 6:**
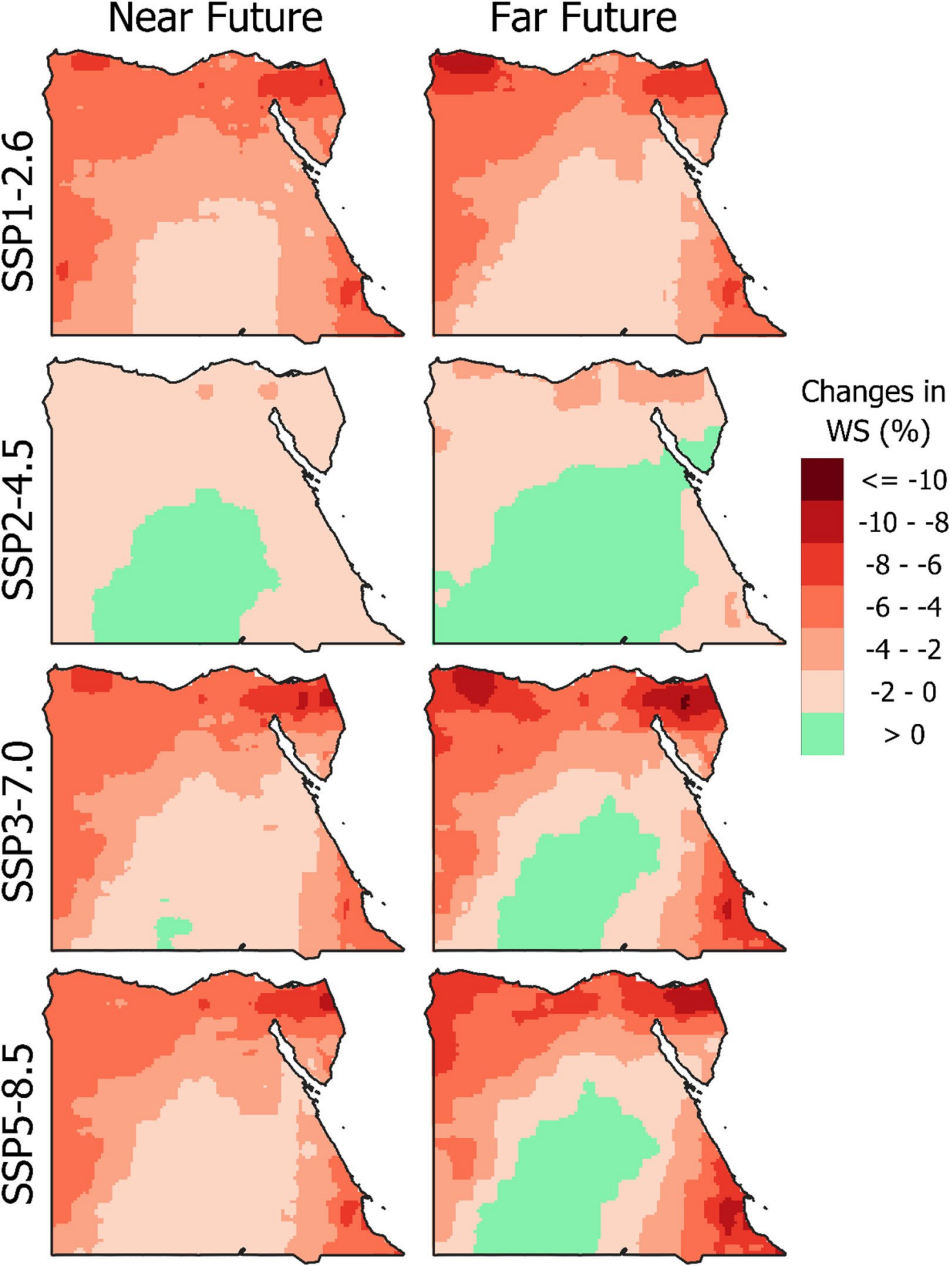
Relative changes (%) in mean MME wind speed for both the near (2020 to 2059) and far future (2060 to 2099) from the historical period.

The relative changes in wind speed for both the near and far future compared to historical values are shown in Fig. 6. Each row shows the relative change in wind speed while applying different SSPs. In the near and far future, the wind speed change while applying SSP1-2.6 is in the range of -6% to 0%. The maximum decrease, in the range of -6% to -4%, is recognised in the north of Egypt. During the far future period, while applying SSP1-2.6, the relative decrease in wind speed reached − 10% to 8% in the north-west of Egypt. While applying SSP2-4.5, a positive change is recognized across the south of Egypt during the near and far future periods. The decrease in the wind speed while applying SSP2-4.5, which reached a maximum decrease of -4%, is considered to be the least compared to the that while applying the other SSPs. In the near future period, the relative change of wind speed is from 0% to -8% while applying SSP3-7.0 and SSP5-8.5. The maximum decrease is in the north which reached − 6% to -8%. During the far future period while applying SSP3-7.0 and SSP5-8.5, although the relative wind speed decreased to more than − 8% in the north, a remarkable positive increase was recognised across the south.

**Fig. 7:**
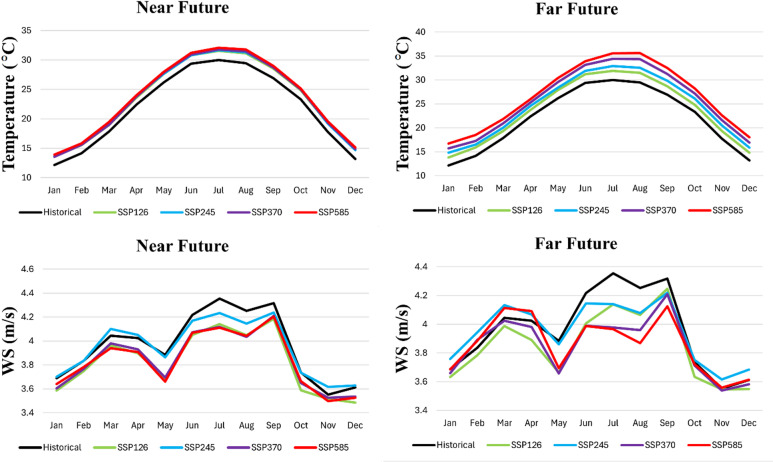
Projected monthly mean temperature (°C) and wind speed (m/s) across the study area in the near (2020 to 2059) and far future (2060 to 2099), comparing the historical baseline against SSP1-2.6, SSP2-4.5, SSP3-7.0, and SSP5-8.5.

Both the monthly temperature and wind speed are presented for the historical, near and far future while considering different SSPs, as shown in Fig. 7. The upper figures compare the historical monthly temperature to the monthly temperature of different SSPs in the near and far future. The historical data are represented by the black line, while the four different SSPs are represented by four different colours. While considering the near future period, it is recognised that the four different SSPs have the same increase. The temperature of the historical and that while applying the four different SSPs in January is in the range from 12.5 to 14 °C and 14.5 to 15.5 °C, respectively. During June and July, a temperature difference of 2 to 3 °C is recognised between the historical and the four different SSPs. Although the temperatures while applying different SSPs in the near future are very close to each other, there is a remarkable variation in the far future for all the temperatures. SSP5-8.5 got the highest temperature of 36 °C during July and August, compared to the other SSPs. The temperature difference while applying SSP1-2.6 and SSP5-8.5 is 4 °C.

The lower figures compare the historical monthly wind speeds to those while considering different SSPs in both the near and far future. In the near future, the pattern of the historical is close to that of the four different SSPs. The historical got the highest wind speed of 4.35 m/s, followed by SSP2-4.5 with a wind speed of 4.25 m/s. During the far future, a remarkable difference is recognised in the pattern of the wind speed from June to September. The historical scenario had the highest wind speeds compared to the other scenarios. The wind speed performance of SSP2-4.5 became close to that of SSP1-2.6 during the period from July to September.

**Fig. 8:**
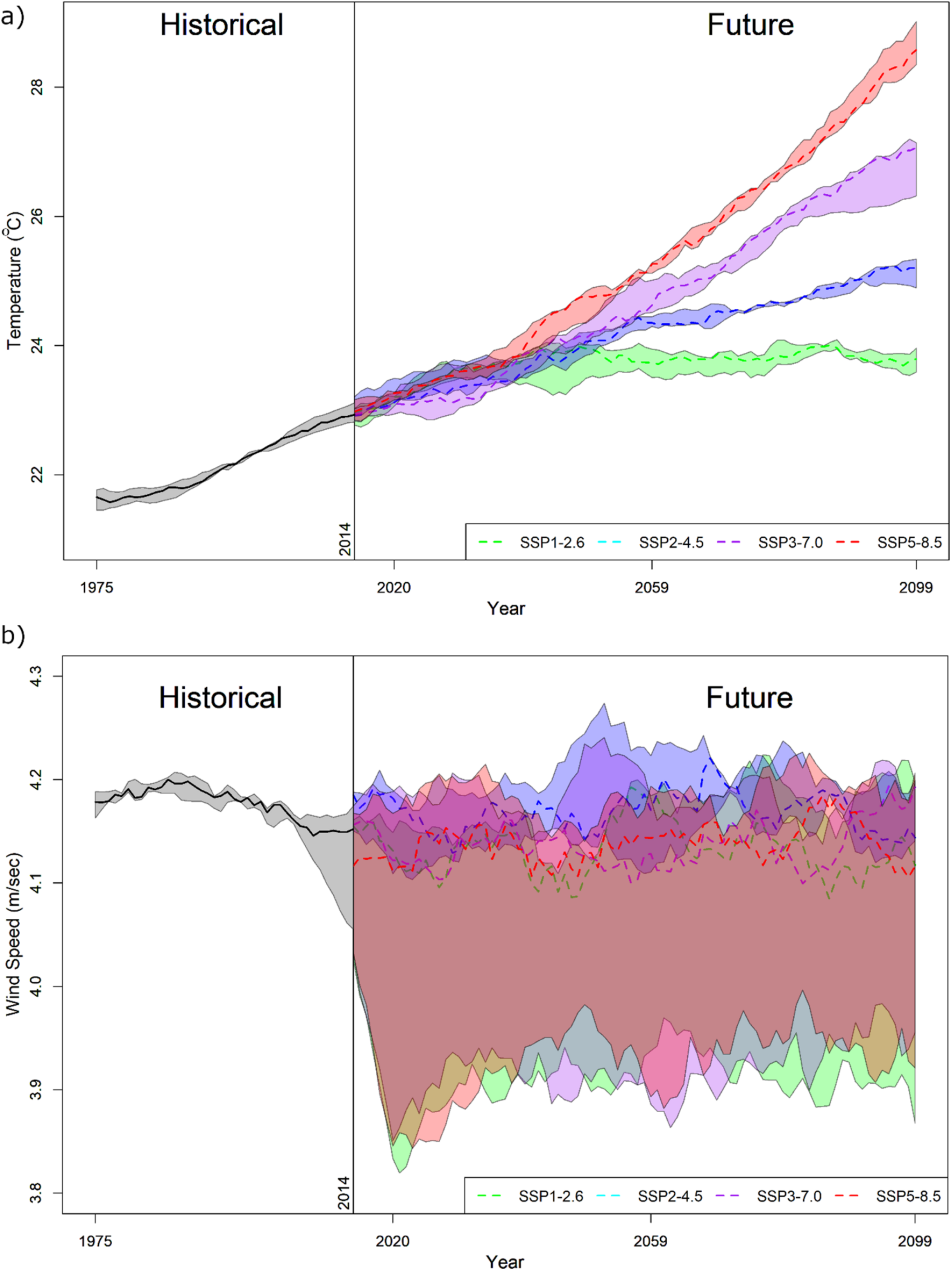
The annual temperature and wind speed in the historical period (1975–2014) represented by the gr*e*y band and future period for SSP1-2.6, SSP2-4.5, SSP3-7.0, and SSP5-8.5. The solid black line and the four dotted colored lines represent the MME mean.

The annual changes during the period from 1975 to 2100 for both the temperature and wind speed are presented in Fig. 8. This period is divided into two periods: the historical and future, from 1975 to 2014 and 2015 to 2100, respectively. The grey band represents the maximum and minimum values of the temperature and wind speed during the historical period, calculated by the three GCMs. Green, blue, purple, and red bands represent the future periods while applying SSP1-2.6, SSP2-4.5, SSP3-7.0, and SSP5-8.5, respectively. The MME mean are represented by a solid black line for the historical and four dotted colored lines for the four different SSPs. The results show an increase in the temperature during the historical and future periods while applying the four different scenarios, as shown in Fig. 8-a. The temperature of the four different scenarios started to increase gradually from 23 °C in 2014. During the period from 2014 to 2100, SSP1-2.6 had the least increase in temperature compared to the other SSPs 1 °C. In contrast, SSP5-8.5 got the highest increase in temperature as it reached 29 °C in 2100. During the period from 2014 to 2100, both SSP2-4.5 and SSP3-7.0 increased by 2 °C and 4 °C, respectively. In contrast to the temperature, there is a decrease in wind speed as shown in Fig. 8-b. During the historical period from 1975 to 2014, the mean wind speed values were in the range from 4.1 m/s to 4.2 m/s. While applying the four different SSPs in the near and far future periods, the wind speeds were in the range from 3.8 m/s to 4.2 m/s. During certain years in the near and far future period, the MME mean wind speed values were lower than 4.1 m/s while applying SSP1-2.6. At the beginning of the far future period, the MME mean wind speed values exceeded 4.2 m/s while applying SSP2-4.5. In 2099, SSP1-2.6 got the lowest MME mean temperature in contrast with SSP5-8.5, which got the highest MME mean temperature value. While considering the mean wind speed in 2099, SSP5-8.5 got the lowest value, and SSP3-7.0 got the highest value.

**Fig. 9:**
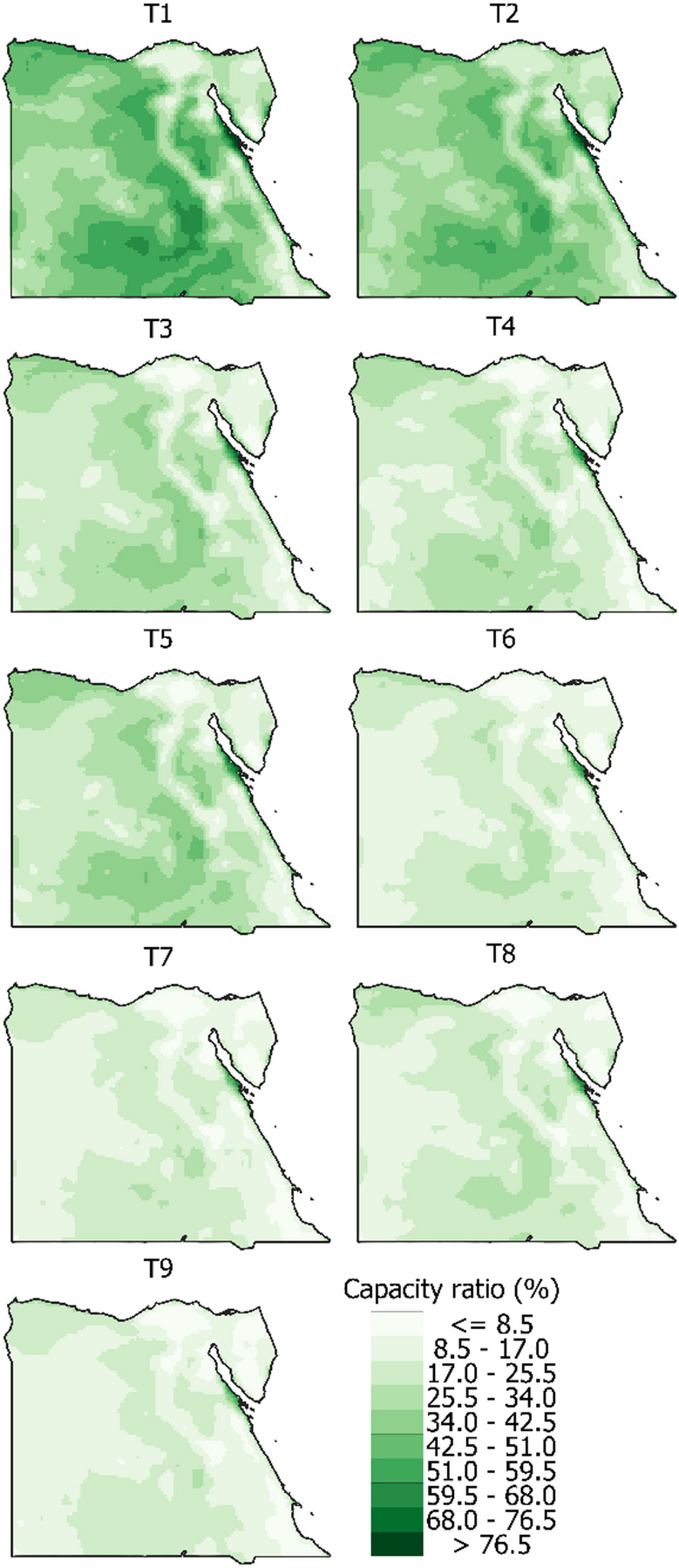
Spatial distribution of the estimated historical capacity ratio (%) for the nine evaluated wind turbine models (T1–T9) across Egypt.

During the period from 1975 to 2014, the predicted output power of the nine wind turbines across Egypt was studied. Three bias-corrected models were used to simulate the climate conditions of both wind speed and temperature. As the wind speed across Egypt does not reach the rated wind speed of the nine wind turbines used in the present study, the capacity ratio of each turbine was calculated as shown in Fig. 9. The capacity ratio is the ratio of the maximum available power to the rated power of the wind turbine. The results show that both wind turbines 1 and 2 are performing better than the other turbines. Wind turbines 1 and 2 reached a maximum capacity ratio across some locations in the range of (68.0 to 76.5%) and (59.5 to 68%), respectively. Wind turbine 9 was considered to have the worst performance compared to the other turbines.

**Fig. 10:**
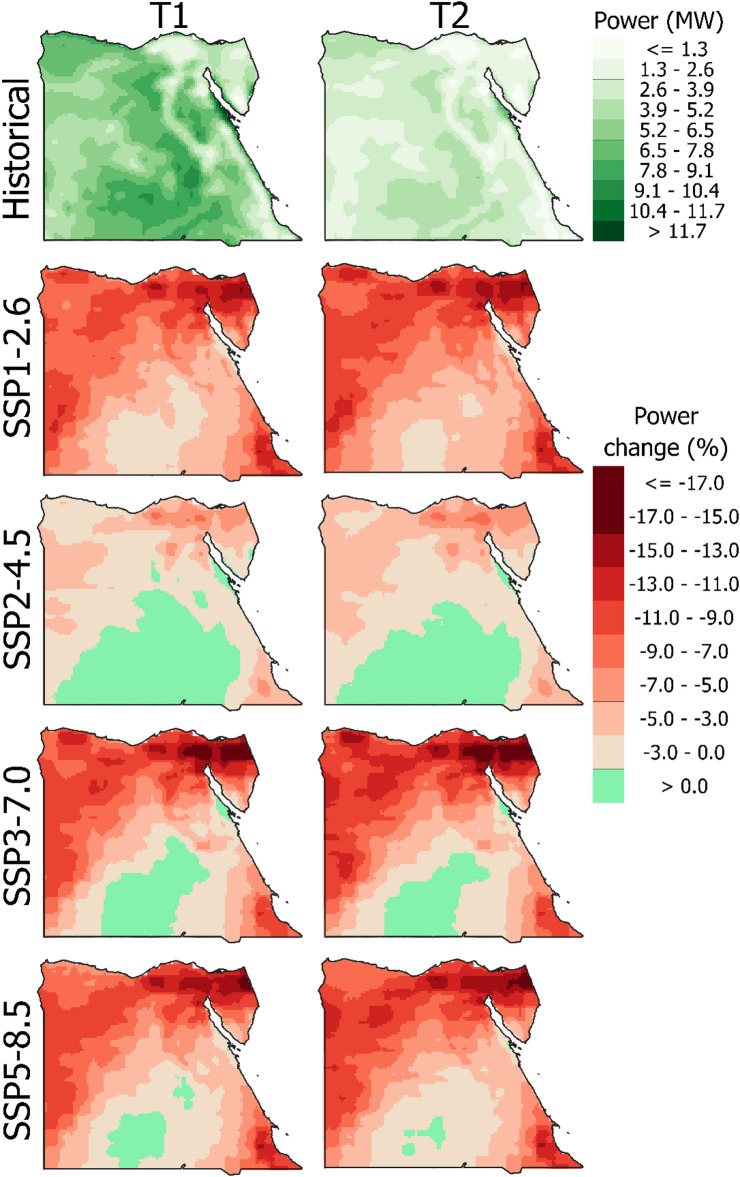
Spatial distribution of historical power output (MW) and projected power change (%) for wind turbine models T1 and T2 across Egypt in the near future. The top row displays the historical power output, while the subsequent rows illustrate the percentage change in power output under SSP1-2.6, SSP2-4.5, SSP3-7.0, and SSP5-8.5.

The output power of wind turbines 1 and 2 during the near future period (2020 to 2059) is shown in Fig. 10. The first row shows the output power while considering the historical data of the wind speed and temperature. In Ras Ghareb, T1 reached an output power of more than 11.7 MW and T2 output power were in the range of 7.8-9.1 MW. The change in power during the near future period is relative to the historical period while applying four different SSPs. SSP1-2.6 exhibits a negative change in power across Egypt compared to the historical data of both T1 and T2. A remarkable decrease in the power is recognized in the North East of Egypt as the change in power was in the range from − 13% to -15%. While applying the other SSPs, there is a positive change across the south of Egypt and in Ras Ghareb. SSP2-4.5 has the best performance, as the positive change is across a large area of Egypt, and the negative change reached − 9% across limited locations.

**Fig. 11:**
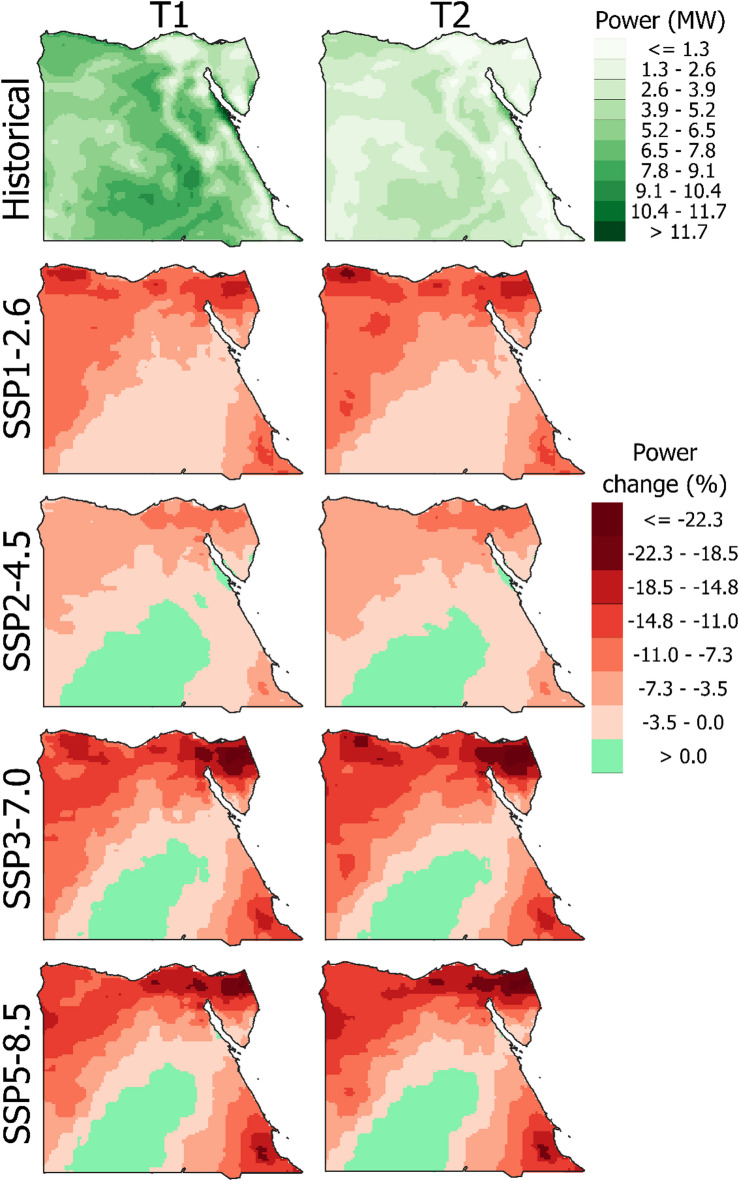
Same as Fig. 10, but for the far future.

The four SSPs from the high-emission pathway SSP5-8.5 to the low-emission pathway SSP1-2.6 were applied to T1 and T2 and compared to a historical baseline in the far future. T1 and T2 are represented in the left and right columns, respectively. The first row shows the output power of T1 and T2 while applying the historical data. Starting from the second row through the fifth row, the change in power is shown while applying SSP1-2.6, SSP2-4.5, SSP3-7.0, and SSP5-8.5, respectively as shown in Fig. 11. While applying SSP1-2.6 there is a negative change in the north east in the range from − 14.8% to -18.5%. In Ras Ghareb, while applying SSP2-4.5 for both T1 and T2, there is a remarkable positive change in the output power of both turbines. A negative change in the power from − 18.5% to -22.3% was recognised while applying SSP3-7.0 and SSP5-8.5 in the north east of Egypt. A positive change is shown clearly in the south of Egypt while applying SSP2-4.5, SSP3-7.0, and SSP5-8.5.

**Fig. 12:**
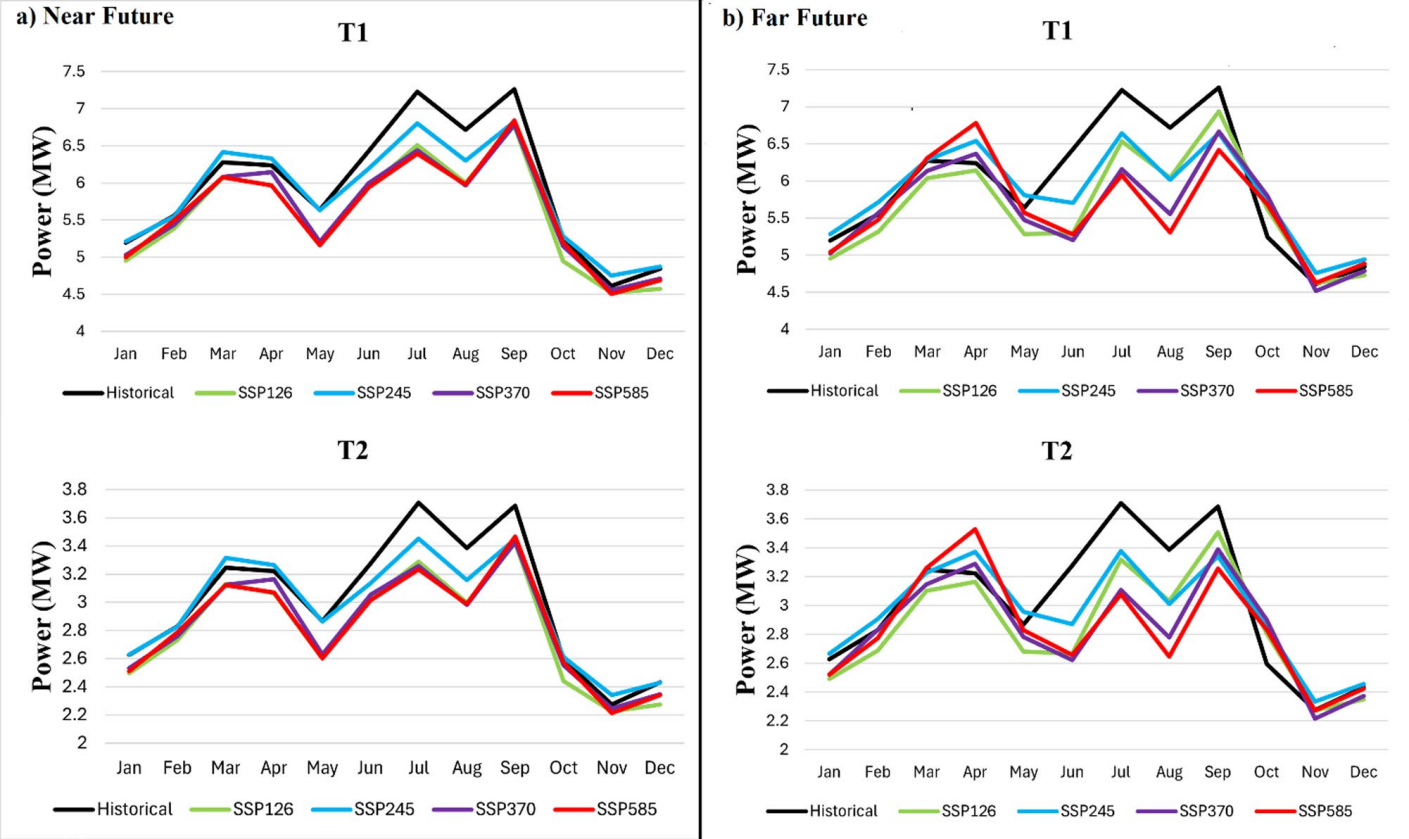
Projected monthly mean power output (MW) for wind turbine models T1 (top row) and T2 (bottom row). The panels compare the historical baseline against SSP1-2.6, SSP2-4.5, SSP3-7.0, and SSP5-8.5 during the (**a**) near future and (**b**) far future periods.

The output power was calculated for the two different wind turbines used in this study while applying the near and far future climate conditions. Figure 12 shows an output power monthly time series. The output power of T1 and T2 is shown in the upper and lower rows, respectively. The right and left columns show the near and far future periods, respectively. The four different SSPs were applied to T1 and T2. Both turbines got the highest output power during July and September. T1 and T2 got the highest output power in SSP2-4.5. While considering the historical and SSP5-8.5 climate conditions to T1, the output power difference is 0.9 and 1.2 MW in the near and far future, respectively. T2 has the same pattern of change as T1. It is observed from this chart that the monthly power output for turbines T1 and T2 exhibits a pattern nearly identical to the wind speed trends shown in Fig. 7, confirming that wind speed variability is the primary driver of the projected energy fluctuations.

**Fig. 13:**
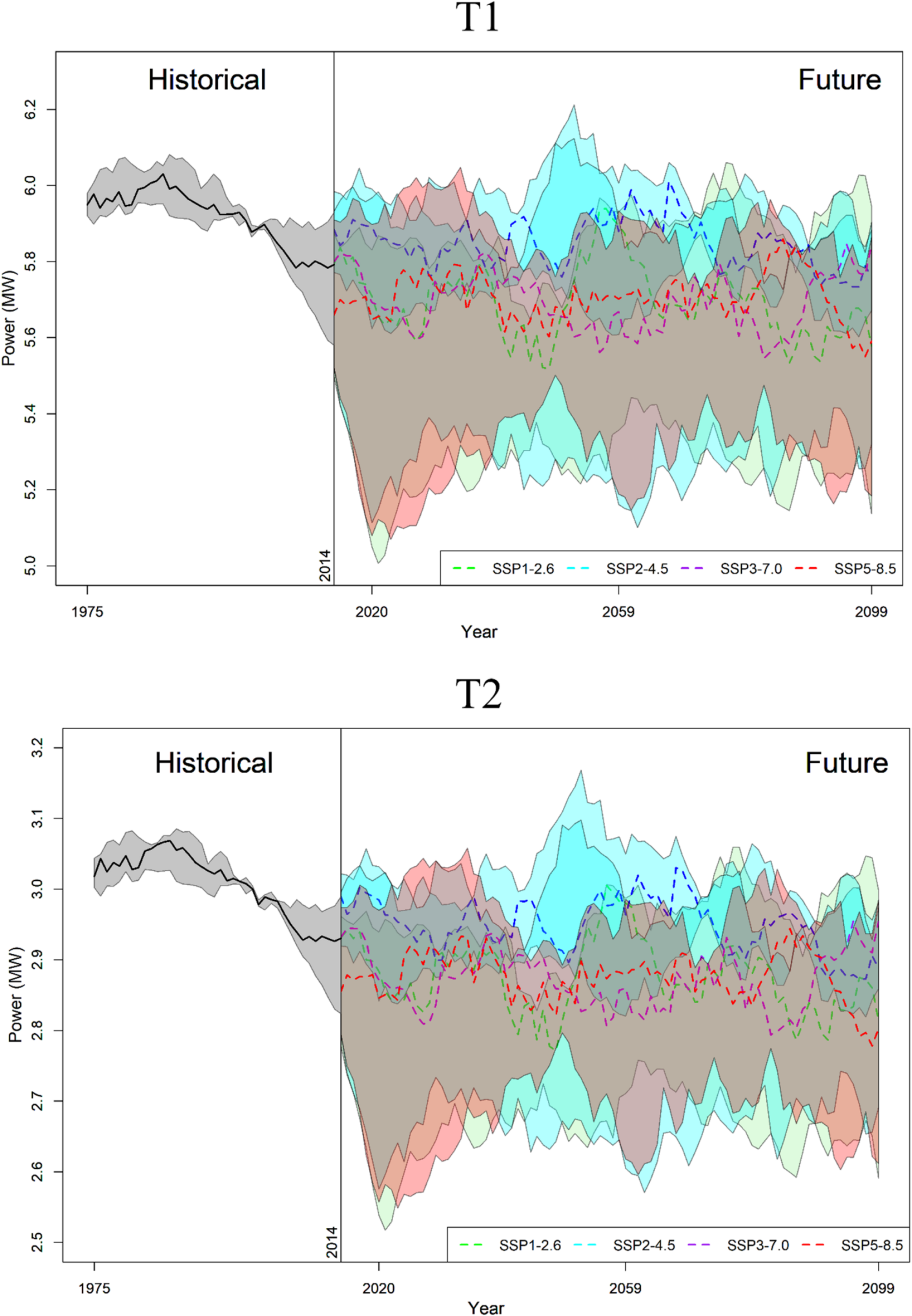
Annual power for T1 and T2 in the historical period (1975–2014) and future period (2015–2099) for the CMIP6 scenarios (SSP1-2.6, SSP2-4.5, SSP3-7.0, and SSP5-8.5).

Based on the annual changes during the period from 1975 to 2100 in both the temperature and wind speed, both wind turbines 1 and 2 were studied while considering these climate conditions, as shown in Fig. 13. This period is divided into two periods: the historical and future, from 1970 to 2014 and 2015 to 2099, respectively. The grey band represents the maximum and minimum output power during the historical period calculated by the four GCMs. Green, blue, purple, and red bands represent the future periods while applying SSP1-2.6, SSP2-4.5, SSP3-7.0, and SSP5-8.5, respectively. The MME means are represented by a solid black line for the historical and four dotted colored lines for the four different SSPs. During the historical period, there is a decrease in the output power for both T1 and T2. T1 output decreased from 6 MW to 5.8 MW by the end of the historical period. The same behaviour was recognized for T2, as the output power decreased from 3 MW to 2.9 MW.

While applying the four different SSPs in the near and far future periods, the output power of T1 and T2 were in the range from 5 MW to 6.1 MW and 2.5 MW to 3.1 MW, respectively. During certain years in the near and far future period, T1 MME mean output power values were lower than 5.5 MW while applying SSP1-2.6. At the beginning of the far future period, the MME mean output power values reached 6 MW while applying SSP2-4.5. After 2014, both turbines got the highest performance while applying SSP2-4.5. By the end of the future period, the output power of both turbines was better when applying SSP3-7.0 than SSP2-4.5. As the same wind speed and temperature were applied to T1 and T2, the same pattern of the output power was recognised for both turbines. In 2099, SSP5-8.5 got the lowest output power MME mean value, while SSP3-7.0 got the highest value.

## Discussion

The findings of this study reveal a detailed and spatially variable impact of climate change on Egypt’s future wind energy potential. Temperature and wind speed of 23 GCMs are evaluated with ERA5-Land data by a single robust statistical metric, KGE, and the best three models are used. The chosen models (EC-Earth3-Veg, EC-Earth3, and CESM2-WACCM) from 1975 to 2100 were bias-corrected to improve their ability to accurately forecast future climatic conditions. This strict selection and calibration resulted in further examination of the efficiency of wind turbines under historical and future contexts, presenting robust evidence in the performance across various SSPs. The central role of temperature and wind speed is in defining the potential of Egypt’s wind energy resources.

Currently, many studies focus on the future of climate change in Egypt^45–49^. The temperature pattern observed in Fig. 5 is strongly supported by recent literature^50^, which predicts an increase in the temperature of 1.5 °C and 1.5-2 °C for the near and far future periods, respectively. Babaeian et al.^51^ have applied the SSP5-8.5 scenario to the Middle East-West Asia, and expected a warming of 2 °C by 2050. The increment in the temperature is recognised not only in Egypt but also in the Middle East^52,53^. Remarkably, these severe warming trends are frequently coupled with alterations in atmospheric circulation that directly impact wind resources^54^. Recent multi-model assessments of the Mediterranean and North African regions project a general reduction in mean wind speeds and cyclone-related wind intensity by the end of the century under high-emission pathways, driven largely by a decrease in regional baroclinicity^55^. Our findings of projected wind power reductions across the majority of Egypt, particularly under the extreme SSP5-8.5 scenario, strongly corroborate this regional literature^56^.

Given Egypt’s northern coastline, evaluating the evolution of offshore wind resources in the Mediterranean Sea through 2100 is essential for a comprehensive energy assessment^35,57–59^. Martinez et al.^60^ evaluated Mediterranean offshore wind resources across three future timeframes (2030–2039, 2060–2069, and 2090–2099) using CMIP6 climate scenarios. Their study identified a decrease in the coefficient of variation in the Eastern Mediterranean under the SSP1-1.9 and SSP5-8.5 scenarios, though this decrease was far less under SSP2-4.5. More critically for long-term energy generation, they projected a widespread decline in mean wind power density throughout the Mediterranean towards the end of the century, particularly under the higher-emission SSP2-4.5 and SSP5-8.5 pathways. These regional findings align closely with the results presented in Fig. 11, which illustrate a projected reduction in wind power output during the far future period.

The simulation results provide a clear indication of how future climate scenarios could influence Egypt’s wind energy potential, particularly when considering the performance of the most efficient turbines, T1 (15 MW) and T2 (10 MW). The moderate mitigation scenario, SSP2-4.5, is projected to result in the highest overall wind power output for T1 and T2, even showing a positive power change in crucial areas like Ras Ghareb and the South of Egypt. This positive change is particularly noteworthy because the majority of Egypt is expected to see a negative change in wind power across all four SSPs. This suggests that under a climate scenario where emissions peak around mid-century and decline, the environmental conditions in key wind farm locations in Egypt remain relatively favourable for these high-capacity turbines compared to other scenarios. The superior performance of SSP2-4.5 over the lower-emission scenario, SSP1-2.6, highlights a complex, non-linear relationship between climate pathway-driven meteorological changes and wind power generation, possibly due to regional wind patterns being more stable or even slightly enhanced in specific geographic hotspots under the intermediate SSP2-4.5 pathway. Several regional and global studies likewise report that SSP2‑4.5 can yield both increases and decreases in wind power density depending on location, with some areas experiencing enhanced resources relative to lower or higher scenarios, reflecting strongly non‑linear circulation responses to climate forcing^61,62^. In particular, projections for the South China Sea^61^ show WPD increases exceeding 10% in all seasons under SSP2‑4.5, while European analyses find that Central Europe^62^ exhibits considerable growth in wind power density in SSP2‑4.5 but reductions under SSP5‑8.5, underscoring that intermediate mitigation can create regional hotspots of relatively favorable future wind conditions.

In contrast, the SSP5-8.5 (very high emissions) scenario is consistently associated with the least favourable conditions, projecting the highest mean temperature (28 °C) and the lowest mean wind speed (3.8 m/sec) by 2100. This extreme warming trend and significant reduction in wind speed are directly linked to the most pronounced negative change in power output for both T1 and T2 across most of Egypt. Even though this high-emission pathway shows a small positive change in power in Ras Ghareb in the far future, the overall annual output remains the lowest among the SSPs, demonstrating the substantial risks of unchecked climate change to the resilience and strategic development of Egypt’s wind energy sector. The consistent meteorological projections of a global warming increase and wind speed decrease by 2100 underscore the criticality of these findings for energy planners and policymakers^59,63^.

This study provides a robust framework for assessing Egypt’s future wind energy potential. The study attempted to minimise error by downscaling all CMIP6 models to the ERA5-Land spatial resolution using bilinear interpolation and selecting the best-performing models using the KGE metric. This ensured that the projections were based on GCMs that most accurately replicate Egypt’s historical climate. Although the wind turbines are consistently getting larger as higher hub heights allow access to faster, more consistent winds, which results in higher capacity factors, the wind turbines used across Egypt are in the medium scale. As the present study focuses on the future of wind energy till 2100, the large-scale wind turbines have been studied under the climate conditions of Egypt. Finally, the study recognises the sensitivity of results to the bias-correction approach. The utilised Quantile Mapping demonstrated exceptional performance by improving the KGE from historical ranges of 0.43–0.55 to approximately 0.99 for the selected GCMs. This ensures the simulated data closely matches the ERA5-Land distribution.

## Conclusion

This study provides a comprehensive assessment of climate change impacts on Egypt’s future wind energy potential using CMIP6 projections. By evaluating turbine performance through the end of the 21st century, the results indicate a widespread decline in future wind power potential across much of the country, driven by rising temperatures under high-emission scenarios (SSP5-8.5). However, under the climate stabilization pathway aligned with SSP2-4.5, specific geographic hubs (southern Egypt and Ras Ghareb) remain notably resilient. In these regions, turbine models T1 and T2 demonstrated maximum output powers of 6 MW and 3.3 MW, respectively.

From a policy and planning perspective, this high spatial variability highlights a crucial need for strategic site selection. Because broader wind potential is expected to decline, developers must prioritize these specific, high-yield regional hubs for future investments. This spatial shift also necessitates proactive grid resilience planning; national transmission infrastructure must be aggressively upgraded to route power from these localized hubs to major demand centres. To build upon these findings, future research should leverage emerging CMIP7 models and machine learning driven bias correction to refine regional projections, while incorporating economic assessments to evaluate the cost-benefit viability of specific turbine deployments under these shifting climatic conditions.

## Data Availability

Data and code will be available upon request from the corresponding author.
